# Development of Small-Diameter Silk Vascular Grafts Supported by Solid-State Nuclear Magnetic Resonance Structural Analysis

**DOI:** 10.3390/molecules30183800

**Published:** 2025-09-18

**Authors:** Tetsuo Asakura, Takashi Tanaka

**Affiliations:** 1 Department of Biotechnology, Tokyo University of Agriculture and Technology, Tokyo 184-8588, Japan; 2 Department of Veterinary Science, Tokyo University of Agriculture and Technology, Tokyo 183-8509, Japan; takashi@go.tuat.ac.jp

**Keywords:** *Bombyx mori* silk fibroin, small-diameter vascular graft, solid-state NMR, structure, silk sponge

## Abstract

This review discusses the development of small-diameter silk-based vascular grafts, based on insights obtained through solid-state NMR structural analysis. With the increasing prevalence of cardiovascular diseases, the demand for vascular grafts with diameters of less than 6 mm is growing. Although synthetic grafts currently used in clinical settings—such as polyethylene terephthalate and expanded polytetrafluoroethylene—are effective, they tend to cause thrombosis and intimal hyperplasia when used as small-diameter vascular grafts. In response to this issue, research has been advancing on new materials that maintain excellent mechanical properties while improving biocompatibility. This review first describes how the detailed structure of silk fibroin (SF) before and after fiber formation was revealed for the first time through solid-state NMR analysis using stable isotope-labeled samples. Then it outlines design criteria for small-diameter SF-based vascular grafts, focusing on fabrication methods like electrospinning. Special attention is given to knitted SF grafts with SF sponge coatings, analyzed via ^13^C solid-state NMR in the dry and hydrated states of the sponges. In vivo performance in rat and canine models is discussed, along with chemically modified SF grafts such as silk-biodegradable polyurethane sponges and their structural and implantation results.

## 1. Introduction

Cardiovascular disease (CVD) is one of the most lethal groups of non-communicable disorders worldwide, causing an estimated 12.1 million to 18.6 million deaths over the past 30 years [1]. Surgical and endovascular approaches, including autologous grafts, remain crucial for treating occluded or narrowed arteries in both large and small vessels. While autologous vascular bypass surgery is commonly used to address arterial disease, it carries postoperative risks such as acute thrombosis, neointimal hyperplasia, and accelerated atherosclerosis, which can lead to graft failure [2]. These complications can significantly impact patients, causing wound infections, discomfort, and prolonged hospital stays, resulting in substantial medical expenses [3,4]. Furthermore, the scarcity of allogeneic donors and challenges in their preservation and manipulation pose additional obstacles to effective CVD treatment. Urgent research into small-diameter artificial vascular grafts and materials is essential to address these critical issues [5].

**Figure 1:**
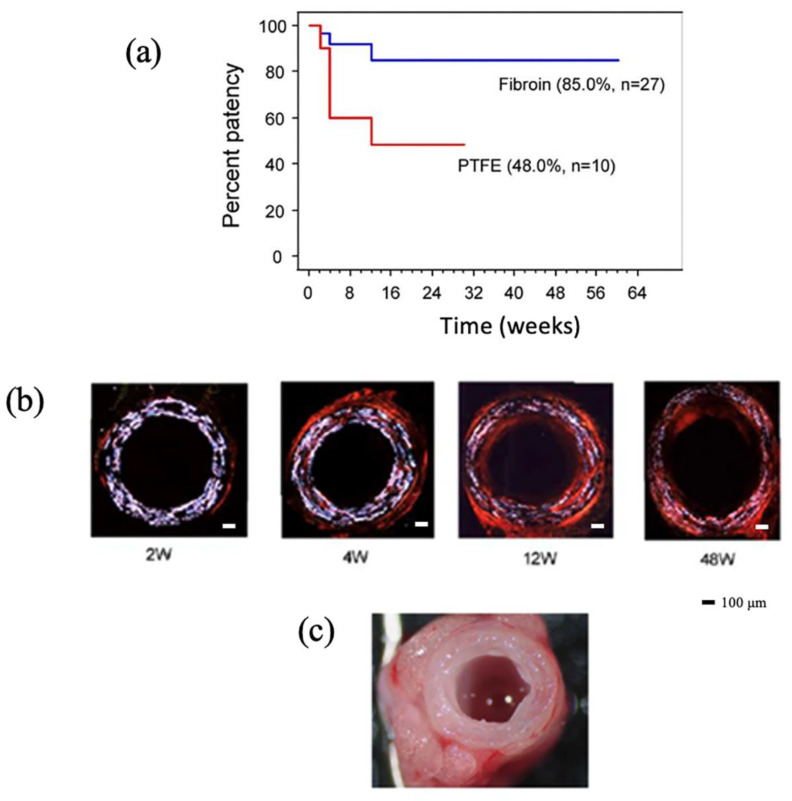
(**a**) Kaplan–Meier analysis showing *B. mori* silk fibroin graft patency of 27 SF and 10 ePTFE grafts implanted into rat aortas at 2, 4, 8, 12, 24, 48, and 72 weeks. (**b**) Polarization microscopic images of the cross-sections of the fibroin grafts after Sirius red staining. The content of fibroin (white) gradually decreased, whereas the collagen (red) content increased from 2 weeks to 48 weeks after implantation. (**c**) Picture of the graft implanted after 1 year. The surrounding tissue appears to be integrated and shows no signs of thrombosis, stenosis, or mechanical failure. Reprinted with permission from [21]. Copyright 2010 Society for Vascular Surgery.

Synthetic polymer materials, such as polyethylene terephthalate and expanded polytetrafluoroethylene, have been used for the preparation of artificial vascular grafts with diameters > 6 mm [6,7]. However, occlusion due to thrombus formation and intimal hyperplasia occurs when these synthetic polymers are used for small-diameter vascular grafts with diameters < 6 mm [8,9,10,11]. Compared with synthetic polymers, natural biopolymers, such as collagen, elastin, and silk fibroin (SF), offer superior cytocompatibility and biocompatibility due to the presence of embedded structural and functional molecules [12]. Among them, SF has several advantages for biomaterials, including the following: 1. High mechanical strength, which can withstand mechanical stress during graft remodeling. 2. An easy-to-prepare SF aqueous solution and the possibility to produce various forms of SF easily. 3. Providing an environment suitable for cell growth while attracting host cells. 4. Minimizing immune response. 5. Moderating biodegradability at the implantation site, which promotes constructive remodeling of the graft [13,14,15,16,17,18,19,20]. For example, Enomoto et al. [21] evaluated the potential of such SF grafts prepared using native silk fibers to generate vascular prostheses for small arteries. Small-diameter SF vascular grafts prepared by plaiting and winding of silk fibers were implanted into rats for an extended time (up to 18 months) and compared with polytetrafluoroethylene (PTFE) grafts. Overall, one-year patency of SF grafts was 85%, which was significantly higher than that of e-PTFE grafts (85 vs. 30%, *p* < 0.01) (Figure 1a). Sirius red staining was used to evaluate biodegradability and extracellular matrix deposition in the SF grafts (Figure 1b). The SF contents decreased gradually until 48 weeks after implantation (33%, *p* < 0.05 at 48 weeks vs. 2 weeks). Conversely, collagen content increased significantly at 12 and 48 weeks. Macroscopic observations of the implanted SF grafts at 1 year confirmed smooth luminal surfaces with no signs of thrombosis or aneurysmal dilation as shown in Figure 1c. Therefore, the results suggest that SF could be a promising material for the development of small-diameter vascular grafts. The advantages of small-diameter SF vascular graft candidates have been reviewed by Thurber et al. [22], Wang et al. [23], Asakura et al. [19], Gupta and Mandal [24], Liu et al. [25], and Settembrini et al. [26].

This review begins by elucidating the molecular and supramolecular structures of SF before and after fiber formation, as characterized by ^13^C solid-state NMR spectroscopy [27] using stable isotope-labeled SF and sequence-specific model peptides. It subsequently outlines the design criteria for the development of small-diameter SF-based vascular grafts, with a focus on fabrication methodologies such as electrospinning. Among these, particular emphasis is placed on knitted SF vascular grafts coated with a silk fibroin sponge, including detailed structural analysis of the sponge via ^13^C solid-state NMR in the dry and hydrated states. The in vivo performance of these grafts following implantation in rat and canine models is also discussed. Furthermore, this review explores the fabrication of small-diameter vascular grafts, incorporating chemically modified SF, notably silk-biodegradable polyurethane sponges, and presents their structural characterization using ^13^C solid-state NMR in the dry and hydrated states. The outcomes of implantation studies in rat models are also reported.

## 2. Silk Fibroin Structure

### 2.1. Primary Structure

**Figure 2:**
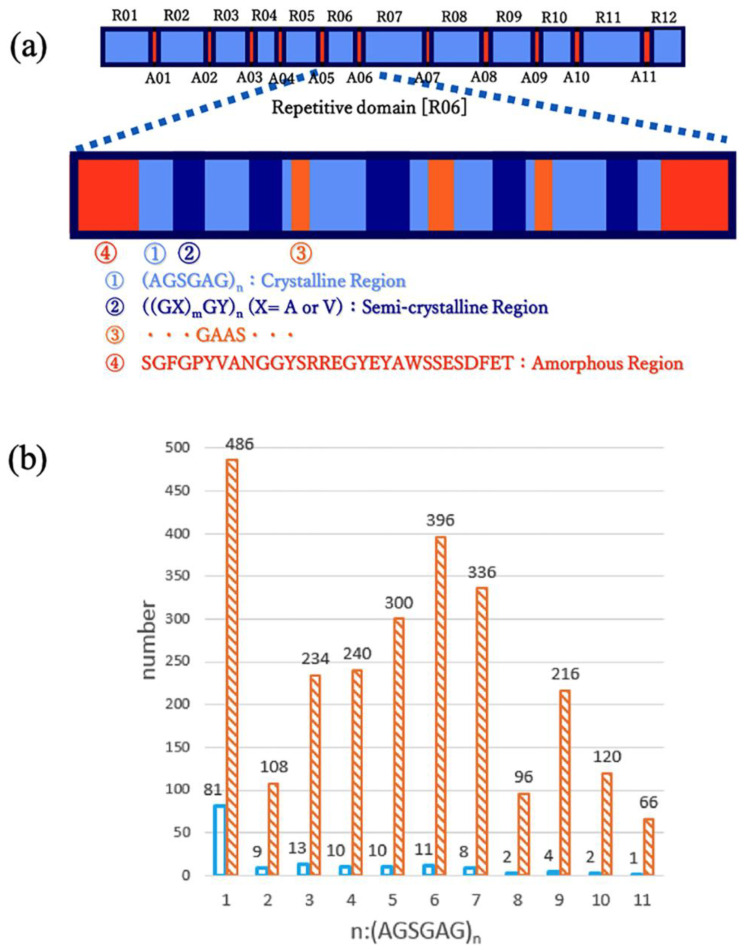
(**a**) The organization of the *B. mori* silk fibroin heavy chain gene. R01, R12, and A01∙A11 represent the predicted twelve repetitive and eleven amorphous regions, respectively, in the polypeptide chain. An approximate amino acid sequence of the R06 region is illustrated by sequences ①–④. (**b**) Histograms of the number of occurrences of each (AGSGAG)_n_ sequence for n = 1–11 (blue), and the total number of amino acid residues of each (AGSGAG)_n_ sequence in the primary structure of the silk fibroin heavy chain (orange stripes). Reprinted with permission from [27]. Copyright 2020 Elsevier.

Silk is composed of two proteins: SF and silk sericin (SS). The SF molecule consists of a heavy (H) chain of 390 kDa and a light (L) chain of 26 kDa connected through a disulfide bond, as well as a glycoprotein P25 (30 kDa) [28,29,30,31,32], and is secreted into the posterior silk gland as an aqueous solution. The H-chain, L-chain, and P25 are considered to be assembled into a high-molecular-mass elementary unit with a ratio of 6:6:1. The H-chain is the primary protein component, making up about 92% of the molecular weight of SF. Its amino acid composition (in mol %) is predominantly Gly (46%), Ala (30%), Ser (12%), Tyr (5.3%), and Val (1.8%) [33]. The primary structure features a repetitive core consisting of alternating arrays of 12 repetitive and 11 amorphous domains, primarily composed of four motifs, as illustrated in Figure 2a [33,34]. Motif ① is a highly repetitive AGSGAG sequence, which constitutes a significant portion of the crystalline domains in SF fibers. This sequence is repeated multiple times, as shown in Figure 2b, with a total of 433 repeats, making up 2598 amino acid residues out of the total 5263 in the H-chain. Thus, nearly half of SF is composed of AGSGAG sequences. Motif ② is a less repetitive sequence containing aromatic and/or hydrophobic residues, mainly Tyr and Val, in sequences like GAGAGY and/or GAGAGVGY, forming the semi-crystalline regions. Motif ③ is similar to motif ① but includes an additional GAAS motif. Motif ④ forms the amorphous regions that separate the domains and contains negatively charged, polar, bulky hydrophobic, and/or aromatic residues, such as TGSSGFGPYVANGGYSGYEYAWSSESDFGT. The properties of SF are essentially derived from the combination of these four motifs, and the unsolved challenge is understanding how these sequence motifs translate into higher-order structures.

### 2.2. Secondary and Higher Order Structure Studied with ^13^C Solid-State NMR

**Figure 3:**
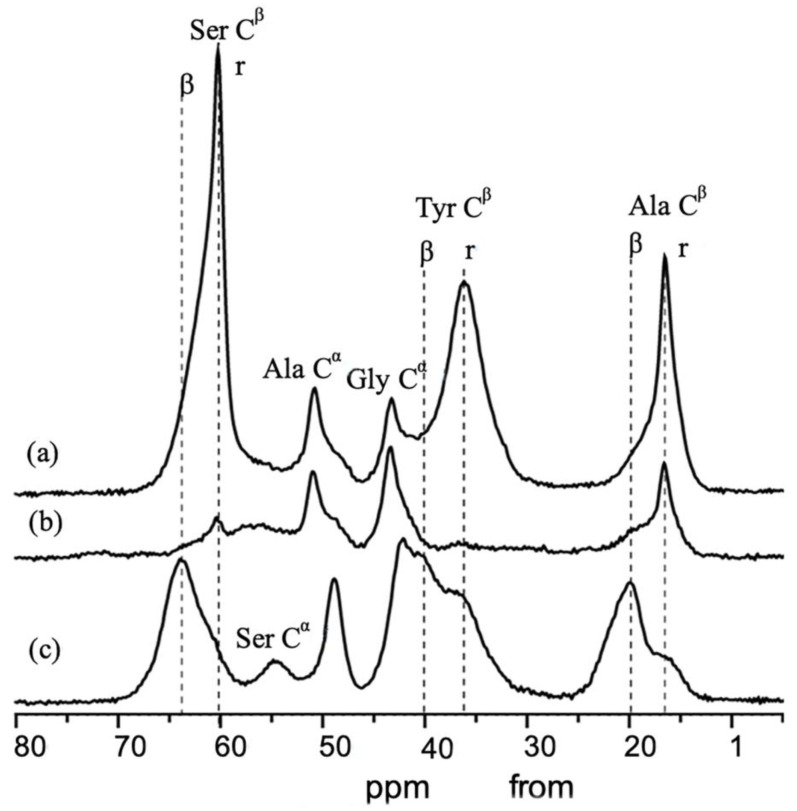
Expanded region (0–80 ppm) in the ^13^C CP/MAS NMR spectra of *B. mori* silk fibroin. (**a**) [3-^13^C]Ser-, [3-^13^C]Tyr-, and [3-^13^C]Ala- silk fibroin with Silk I form before fiber formation. (**b**) Non-labeled silk fibroin with Silk I form before fiber formation and (**c**) [3-^13^C]Ser-, [3-^13^C]Tyr-, and [3-^13^C]Ala- silk fibroin fiber with Silk II form. r: Random coil and b: b-sheet structure. Reprinted with permission from [36].

**Figure 4:**
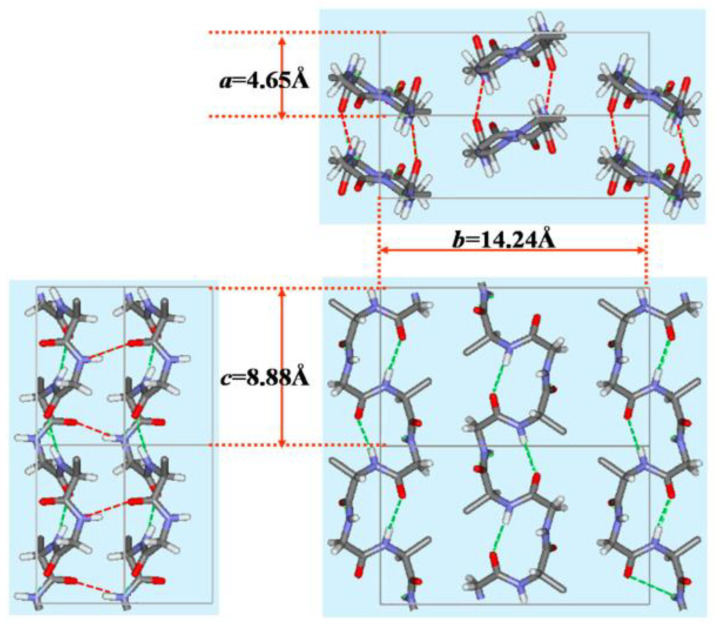
Packing structure of poly(Ala-Gly) chains with type II β-turn structure as a model for Silk I*, shown in three orthogonal directions. Dotted lines denote intra- (green) and inter- (red) molecular hydrogen bonds. The unit lattice values ***a***, ***b,*** and ***c*** were obtained from X-ray diffraction data. Reprinted with permission from [39].

Two crystalline forms, Silk I and Silk II, have been proposed as polymorphs of SF [35]. Silk I represents the solid-state structure of SF before spinning. The sample was extracted from the middle silk glands under mild conditions and subsequently dried. The ^13^C cross-polarization/magic angle spinning (CP/MAS) NMR spectra of Silk I are shown in Figure 3a,b, where ^13^C labeling of Ser and Tyr Cβ carbons was performed for the sample of Figure 3a [36]. Because of ^13^C labeling, the conformational analysis can be obtained for Ser and Tyr residues. Conversely, the solid-state structure of SF after spinning is referred to as Silk II, and its ^13^C CP/MAS NMR spectrum is depicted in Figure 3c, with ^13^C labeling of Ser and Tyr Cβ carbons. The main ordered solid-state conformation of Silk I is named as Silk I*, which is adopted by regular repeat sequences such as motif ① [37]. This conformation is a repeated type II β-turn (Figure 4) [37,38,39,40]. The structure was determined using a range of solid-state NMR techniques, with a combination of stable isotope-labeled SF and model peptides, in particular (AG)_15_, which is a good model for the typical sequence of the crystalline domain of SF.

**Figure 5:**
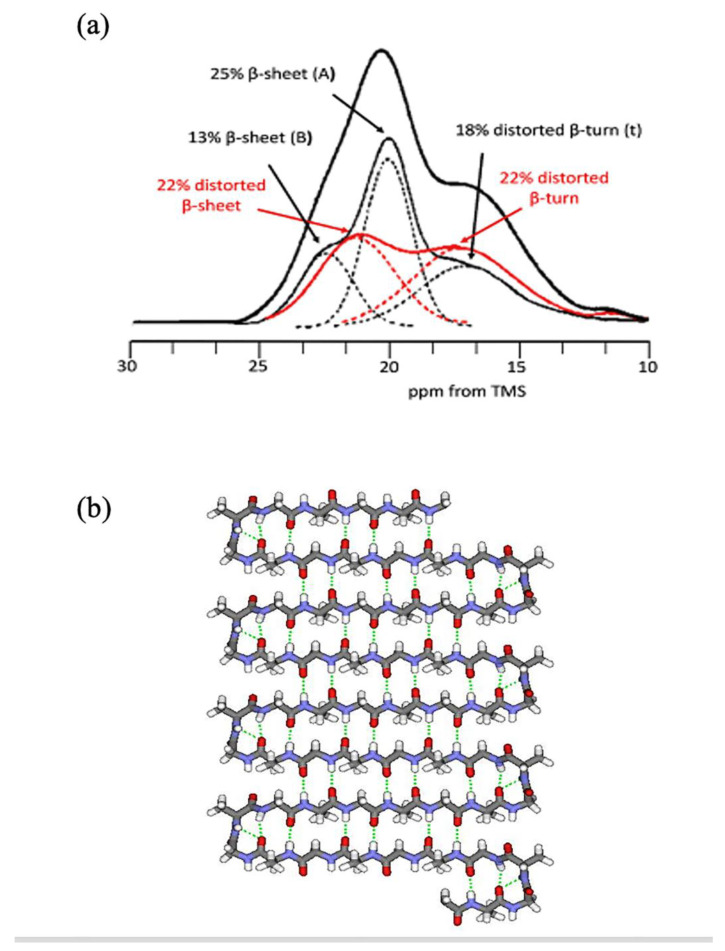
(**a**) Expanded Ala Cβ peak from the ^13^C solid-state NMR spectrum of [3-^13^C]Ala *B. mori* silk fibroin fiber (thick solid, black) with the assignment of each component. The crystalline domain (56%; thin solid, black) with the sequence (AGSGAG)_n_ consists of 18% distorted β-turns (t), 25% β-sheet (A), and 13% β-sheet (B). The non-crystalline domain (44%, red) consists of 22% distorted β-turns and 22% distorted β-sheets. Reprinted with permission from [46]. (**b**) The 8-residue periodic lamellar structure (Silk II) of the crystalline part of *B. mori* silkworm silk. Reprinted with permission from [48].

On the other hand, a model of Silk II was first proposed by Marsh, Corey, and Pauling [41] using X-ray fiber diffraction. Their model was an antiparallel (AP) β-sheet with the side chains in a polar arrangement. However, subsequent studies using X-ray diffraction [35,42,43,44] showed a less regular structure than proposed by this model, although the AP β-sheet structure was accepted. Later, the solid-state NMR was used to determine the Silk II structure [45,46,47,48,49,50,51]. Figure 5a shows the ^13^C CP/MAS NMR spectrum of the Ala Cβ carbons of native [3-^13^C]Ala-SF fibers with Silk II form [46]. The spectrum could be deconvoluted into two components, from the crystalline (56%; thin solid, black) with the sequence (AGSGAG)_n_, which consists of 18% distorted β-turns at 16.5 ppm, 25% β-sheet at 19.5 ppm (A), and 13% β-sheet at 22.1 ppm (B). The non-crystalline domain (44%, red) consists of 22% distorted β-turns and 22% distorted β-sheets. The crystalline component could be further interpreted with an antipolar lamellar structure with repetitive folding through β-turns every eighth amino acid (Figure 5b). Further details regarding the structural determination are available in the references [47,48,49,50,51].

## 3. Requirements for the Fabrication of Small-Diameter Silk Fibroin Vascular Grafts

**Figure 6:**
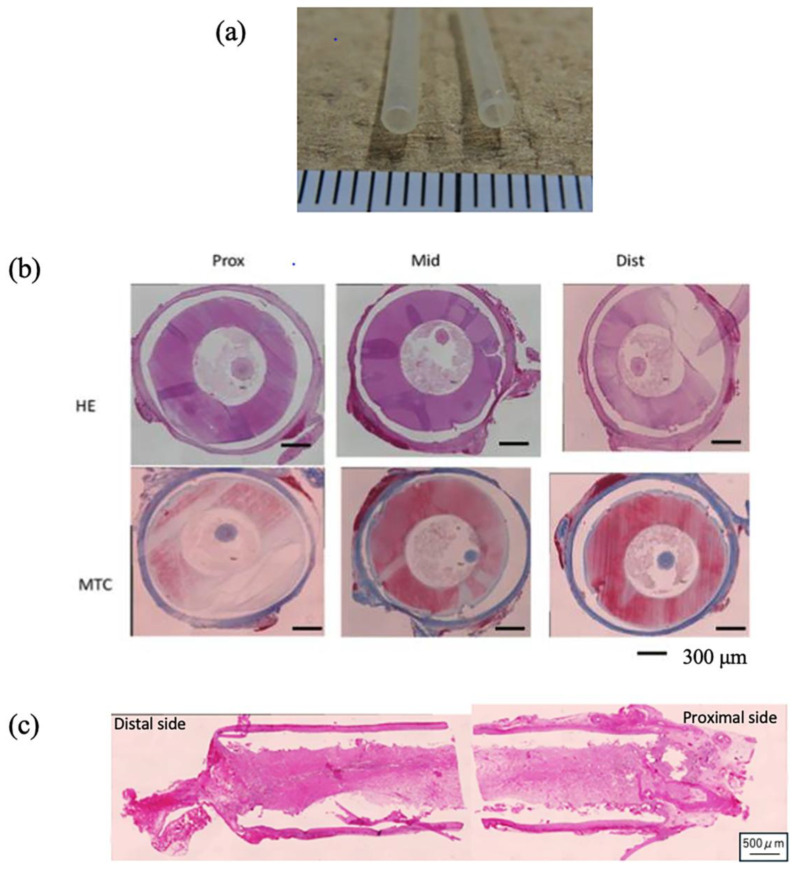
(**a**) Macroscopic image of shrinkage of *B. mori* silk fibroin vascular grafts before implantation. (**b**) Hematoxylin and Eosin (HE) and Masson’s Trichrome (MTC)-stained cross-sections of shrinkage of silk fibroin vascular graft after 4 weeks of implantation. The part of the cross-section is proximal (Prox), middle (Mid), and distal (Dist). (**c**) Longitudinal section and HE stain results of the silk non-porous coating small-diameter silk fibroin vascular graft after 4 weeks of implantation.

SF is a well-established natural biomaterial with a long-standing history of clinical use, particularly in surgical sutures [13,14,52,53]. As a biomaterial, SF offers several advantageous properties: 1. exceptional biocompatibility; 2. tunable biodegradation rates; 3. low immunogenicity; 4. superior mechanical properties; 5. high versatility in processing [54,55,56], enabling fabrication into diverse scaffold forms such as films [57,58], fibers [59], porous sponges [60,61], and hydrogels [62]; 6. abundant availability; 7. cost-efficiency; and 8. environmentally friendly processing methods [13,63]. Silk-based materials have been applied in the regeneration of various tissues, including bone, cartilage, vasculature, skin, ocular tissues, and nerves [64,65,66,67,68,69]. Although SF is biodegradable in vivo, its degradation proceeds relatively slowly, which can delay the replacement of the scaffold with native tissue [70]. To address this, tubular silk scaffolds with enhanced degradability were developed using a gel contraction technique under mild conditions, as illustrated in Figure 6a, and subsequently implanted into rats [71].

These small-diameter SF vascular grafts, however, were rigid, complicating surgical anastomosis. Despite this, no immediate blood leakage or occlusion was observed post-implantation. Pulsation was detectable distal to the graft site, albeit weak, and the grafts appeared significantly thinner than native vessels upon gross examination. Histological results are presented in Figure 6b. Four weeks post-implantation into the rat abdominal aorta, nearly complete occlusion was observed, with no evidence of tissue ingrowth into the graft wall. This outcome is likely due to the graft’s stiffness and lack of elasticity, resulting in a compliance mismatch with the host vessel. Furthermore, the non-porous nature of the graft hindered cell adhesion and prevented material exchange across the graft wall, impeding tissue integration [72,73]. Such compliance mismatches are known to contribute to long-term complications like intimal hyperplasia, which can lead to stenosis and eventual occlusion. Additionally, the absence of endothelial lining—critical for preventing thrombosis and hyperplasia—posed a challenge. Endothelialization typically occurs via migration from the anastomosis site, circulating progenitor cells, or neovascularization within the graft. In this case, early remodeling did not occur, likely due to these limitations. Figure 6c shows similar results for non-porous SF-coated grafts, with near-total occlusion after four weeks. These findings underscore the importance of incorporating porosity and flexibility into vascular graft design to promote integration and function, highlighting the need for further refinement.

## 4. Various Small-Diameter Silk Fibroin Vascular Grafts

SF aqueous solutions are typically prepared by removing SS from silk cocoons via soap-based degumming, though enzymatic or alkaline methods are also used. The degummed SF fibers are dissolved in heated neutral salts like lithium bromide or calcium chloride, then dialyzed against distilled water to obtain a purified SF solution. Drying this solution forms SF films, which can be shaped into tubular structures using dipping techniques for vascular graft applications. In a study by Lovett et al. [73], silk microtube porosity was tuned using poly(ethylene oxide). SF microtubes with inner diameters from 0.1 to 6.0 mm were fabricated by dipping stainless steel wires into the SF solution. These tubes withstood physiological pressures and supported protein diffusion and cell infiltration.

**Figure 7:**
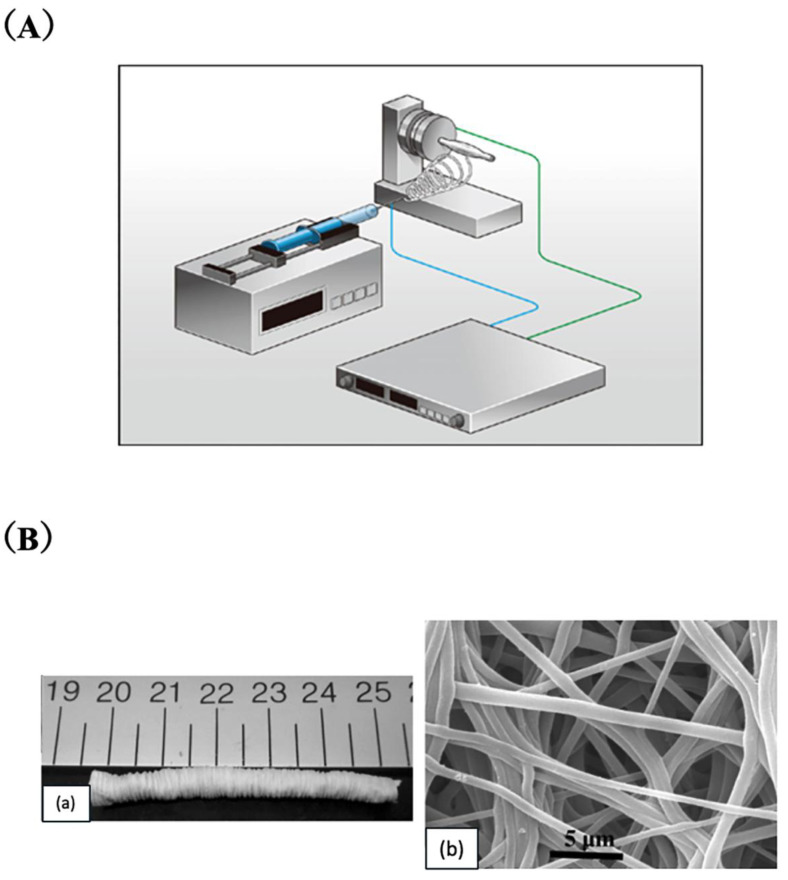
(**A**) A series of electrospinning apparatuses to prepare small-diameter SF vascular grafts. The grafts can be obtained from the accumulation of SF fibers deposited on the rod. Reprinted with permission from [19]. (**B**) (**a**) Microphotograph of an electrospun SF tube (cm). (**b**) SEM micrograph of the inner surface of the electrospun SF tube. Micrographs taken at 15 keV and 10 mA. Reprinted with permission from [78]. Copyright 2010 Elsevier.

Electrospinning, which uses high-voltage fields to produce nano/microfibers from polymer solutions, is widely used in SF-based vascular grafts. The electrospinning apparatus to prepare small-diameter SF vascular grafts is shown in Figure 7A [19].

Zhang et al. [74] studied Human Aortic Endothelial Cells (HAECs) and Human Coronary Artery Smooth Muscle Cells (HCASMCs) on electrospun SF scaffolds, observing HCASMC alignment on random fibers and HAECs forming cord-like structures. A capillary-like network with visible lumens indicated strong cell–matrix interactions. Building on this, Zhang et al. [75] developed small-diameter SF grafts by sequentially seeding HCASMCs and HAECs onto tubular scaffolds, cultured under flow in a dual-loop bioreactor. This dynamic environment enhanced cell alignment, extracellular matrix (ECM) deposition, and physiological phenotypes. Electrospun 3D SF scaffolds also showed high porosity and strong mechanical properties. For example, Zhou et al. [76] and Soffer et al. [77] developed electrospun SF tubes capable of withstanding arterial pressures and mimicking the mechanical behavior of native vessels. These properties can be fine-tuned by adjusting fiber orientation and electrospinning parameters. To achieve the high concentrations required for electrospinning, SF is often dissolved in organic solvents. Tubular scaffolds electrospun from SF dissolved in formic acid, shown in Figure 7B, have demonstrated compatibility with both human saphenous veins and rat abdominal aortas [78]. This is critical, as mismatched mechanical compliance is a major contributor to intimal hyperplasia in small-diameter tissue-engineered vascular grafts (TEVGs) [79]. Hexafluoro-isopropanol (HFIP) is another solvent used for SF electrospinning [80], producing scaffolds with superior mechanical strength, faster endothelialization, more M2 macrophages, and a stable medial layer compared to water-based methods [81]. In rat aorta implants, HFIP-spun SF grafts showed host cell infiltration, new tissue and elastic layer formation, and surface vascularization [82]. A 24-week study comparing SF and ePTFE grafts found higher survival with SF (95% vs. 73%) and complete endothelial coverage by week 6, along with neointima regression, indicating an SMC phenotype shift [83]. To enhance porosity without compromising strength, air-impedance electrospinning was developed using a pressurized perforated mandrel [84].

Recent preclinical studies tested electrospun poly(L-lactide-co-ε-caprolactone) (PLCL) grafts coated with silk/heparin gel in rabbit carotid arteries for up to eight months [85]. A three-layer SF/polyurethane (PU) composite graft was also evaluated as an arteriovenous shunt in sheep, maintaining 100% patency over 90 days [86,87]. Electrospinning remains a leading method for TEVG fabrication, offering a high surface area via nanofibers, though low porosity remains a challenge addressed by various innovations [88].

To further enhance mechanical strength and water permeability, Sato et al. [89] developed small-diameter SF grafts by coating electrospun SF tubes with an SF sponge. This modification significantly improved both circumferential and longitudinal tensile strength and elasticity compared to uncoated grafts. A comparable study was conducted by Alkazemil et al. [90], where the integration of an SF sponge layer into electrospun SF tubes led to a reduction in water permeability. However, the permeability remained within a range conducive to endothelial cell growth [91]. Beyond electrospinning, various fabrication techniques have been employed to produce three-dimensional porous SF scaffolds. These include freeze-drying [92,93], salt leaching [94,95], freeze–thaw cycles [96], electrohydrodynamic bubbling [97], rapid prototyping [98], and simpler methods utilizing n-butanol [99]. Porous scaffolds provide a 3D matrix that supports cell seeding, growth, and vascularization. Tubular vascular scaffolds can be fabricated via molding, dipping, electrospinning, knitting, or gel spinning. Gel spinning offers precise control over structure, including winding patterns and pore size. Post-processing methods like methanol treatment, air drying, and freeze-drying help optimize mechanical and biological properties. Lovett et al. [100] showed that gel-spun SF tubes have strong mechanical properties and support HCASMC and Human Umbilical Vein Endothelial Cell (HUVEC) adhesion, confirming biocompatibility. They later used freeze-dried SF grafts with a porous, layered structure to regulate vascular cell behavior [101]. Implanted in rat aortas, these grafts matched native vessel flexibility and supported endothelial lining. Liu et al. [102] developed a bilayer SF scaffold with a woven, heparin-treated inner layer and a porous, ECM-like outer layer, offering excellent hemocompatibility and mechanical resilience. Textile-engineered SF scaffolds also provide superior flexibility and compliance, making SF-based vascular grafts a key focus in biomedical research.

## 5. Silk Knitted Small-Diameter Vascular Graft Coated with Silk Fibroin Sponge

At present, four primary textile-based techniques are utilized in the fabrication of artificial vascular grafts: warp knitting, weaving, braiding, and electrospinning [103,104]. Woven grafts are known for their outstanding mechanical strength and dimensional consistency, while knitted structures offer superior flexibility, elasticity, and porosity. Braided constructs provide excellent resilience and compressive strength, and electrospun scaffolds, as discussed in the previous section, offer significant biological benefits. By adjusting structural design parameters—such as increasing the number of floating warp threads, altering the braiding angle, or reducing the fiber thickness and diameter—engineers can achieve optimal porosity and mechanical robustness. In addition, a variety of physical, chemical, and biological surface modification strategies can be employed to enhance graft performance, including improvements in biocompatibility, antibacterial activity, anti-inflammatory response, and endothelial cell integration. This review emphasizes the development of SF-based vascular grafts produced using warp knitting methods, further enhanced by coating with SF sponge layers [104,105,106,107,108,109,110,111,112,113,114,115,116].

### 5.1. Selection of Knitting Methods to Prepare Small-Diameter Silk Vascular Graft Bases Using the Double-Raschel Knitting Technique

Knitting is a textile technique that creates fabric by interlocking loops of yarn, resulting in a softer and more pliable material compared to woven textiles. There are two main categories of knitted fabrics: warp knitting and weft knitting [104]. In warp knitting, yarns are fed in the longitudinal (warp) direction and systematically looped over individual needles on a warp knitting machine, forming the fabric in a coordinated manner. Conversely, weft knitting involves feeding multiple yarns in the transverse (weft) direction, which are simultaneously looped over needles to produce fabric on a weft knitting machine. Weft-knitted meshes tend to unravel easily and are more susceptible to yarn displacement, whereas warp-knitted fabrics offer superior elasticity and multidirectional stretch. These fabrics can be produced in various forms, ranging from thin sheets to complex three-dimensional structures, and their edges remain stable when cut, making them particularly suitable for vascular graft applications.

The use of double-raschel warp knitting is especially advantageous in this context. This method ensures that silk threads maintain their round profile even under pressure from guides or needles, preserving their elasticity. Additionally, the dense network of contact points between fibers contributes to enhanced mechanical strength and prevents edge fraying during surgical implantation [117]. Reduced friction at these contact points also minimizes the risk of thread breakage or separation during production.

**Figure 8:**
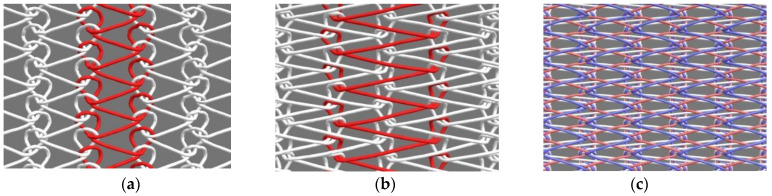
Structures of (**a**) single tricot, (**b**) single code, and (**c**) double tricot knittings prepared using a double-raschel warp knitting machine.

**Figure 9:**
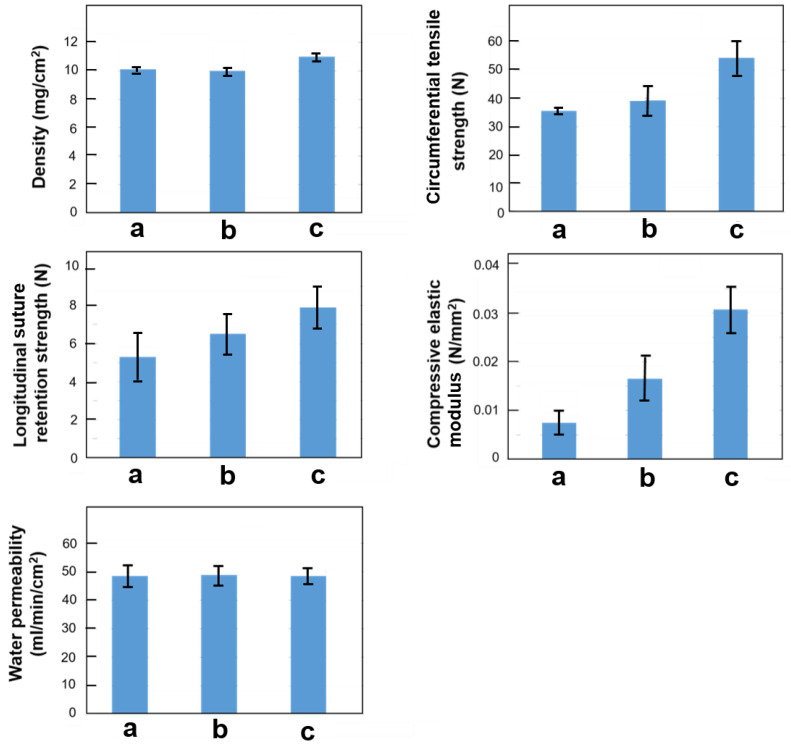
Density, circumferential tensile strength, longitudinal suture retention strength, compressive elastic modulus, and water permeability of knitted silk vascular grafts with three structures of (a) single tricot, (b) single code, and (c) double tricot knittings. Density measurements were conducted using only the silk vascular graft base, while all other samples consisted of silk vascular grafts coated exclusively with silk fibroin [21]. Among the three structures, the silk vascular graft with a double tricot knitted structure showed the highest density, leading to superior tensile strength, compressive modulus, and suture retention. These features address common issues in knitted grafts, like mechanical weakness and edge fraying. Water permeability was similar across all types, making the double tricot method the most balanced in terms of mechanical performance and structural integrity. In fact, silk vascular grafts with single code knitting and double tricot knitted structures, coated with a sponge using PGDE as a porogen, were implanted into dogs. Two months later, echo Doppler imaging revealed that the graft with the single code knitting had expanded more than 1.5 times, whereas the silk graft with the double tricot knitted structure had barely expanded.

Notably, the double-raschel knitting technique has already been applied in the commercial fabrication of polyester-based vascular grafts [105]. Figure 8 illustrates three types of warp-knitted silk vascular graft structures produced using a double-raschel warp knitting machine: (a) single tricot, (b) single code, and (c) double tricot configurations. Figure 9 presents a histogram comparing the physical and mechanical properties—namely density, circumferential tensile strength, longitudinal suture retention strength, compressive elastic modulus, and water permeability—of silk vascular graft substrates fabricated using three different warp-knitted structures [106,107]. Density measurements were conducted using only the silk vascular graft base. On the other hand, the other samples were prepared by immersing the silk vascular graft bases in a silk aqueous solution only, followed by freeze-drying to coat them with silk. Subsequently, the samples were immersed in alcohol to render the silk insoluble, resulting in insolubilized silk-coated silk vascular grafts, which were used for physical property measurements. Among the three, the silk vascular graft with a double tricot knitted structure exhibited the highest density, which contributed to its superior circumferential tensile strength and compressive modulus. These characteristics are particularly valuable, as knitted grafts often face challenges related to mechanical weakness and limited dimensional stability. Additionally, the double tricot configuration demonstrated excellent longitudinal suture retention strength, an important factor in preventing edge fraying when sutures are applied during surgical implantation. In contrast, water permeability levels were found to be relatively consistent across all three knitting types. In fact, silk vascular grafts with single code knitting and double tricot knitted structures, coated with a sponge using PGDE as a porogen, were implanted into dogs. Two months later, echo Doppler imaging revealed that the diameter of the graft with the single code knitting had expanded more than 1.5 times, whereas the silk graft with the double tricot knitted structure had barely expanded. Taken together, these findings suggest that the double tricot knitting method offers the most favorable balance of mechanical performance and structural integrity among the evaluated designs.

### 5.2. Preparation of Silk Sponge and Structural Analysis by ^13^C Solid-State NMR

SF is recognized as a highly promising biomaterial due to its outstanding mechanical strength, excellent biocompatibility, and versatility in being processed into various formats such as fibers, powders, films, gels, and sponges. Among these, SF sponges are particularly valuable for biomedical applications, including corneal regeneration scaffolds, bone repair matrices, wound dressings, and coatings for vascular grafts.

Typically, SF sponges are produced by freeze-drying concentrated SF aqueous solutions. However, these sponges are water-soluble and require stabilization through insolubilization treatments. Alcohols like methanol are commonly used for the purpose mentioned above, but they tend to increase the stiffness of the sponge, slow down its biodegradation, and create mechanical mismatches with soft tissues such as skin and muscle.

Studies by Lu et al. [118] and Pei et al. [119] have shown that treating SF sponges with glycerin (Glyc)—a known plasticizer and humectant—results in improved biocompatibility and softer mechanical characteristics, making them more suitable for tissue engineering. Glyc has thus emerged as an effective agent for insolubilizing SF. Structural changes induced by Glyc in SF films have been investigated using analytical techniques such as FT-IR spectroscopy, XRD, DSC, DMTA, TGA, fast scanning calorimetry, and solid-state NMR. These studies revealed that Glyc-treated SF films contain fewer β-sheet structures compared to methanol-treated ones, contributing to their enhanced flexibility. Moreover, the Silk I* conformation (Type II β-turn) has been identified in Glyc-treated films using ^1^H Double-Quantum Magic Angle Spinning (DQMAS) NMR spectroscopy. Min et al. [120] developed porous tubular SF sponges using poly(ethylene glycol) diglycidyl ether (PGDE) as a porogen. These sponges are transparent and flexible when hydrated and exhibit excellent mechanical resilience, particularly in terms of deformation recovery. PGDE can be completely removed by soaking the composite films in water for three days. Consequently, SF sponges fabricated using Glyc or PGDE as porogens show great promise as scaffolding materials in regenerative medicine.

**Figure 10:**
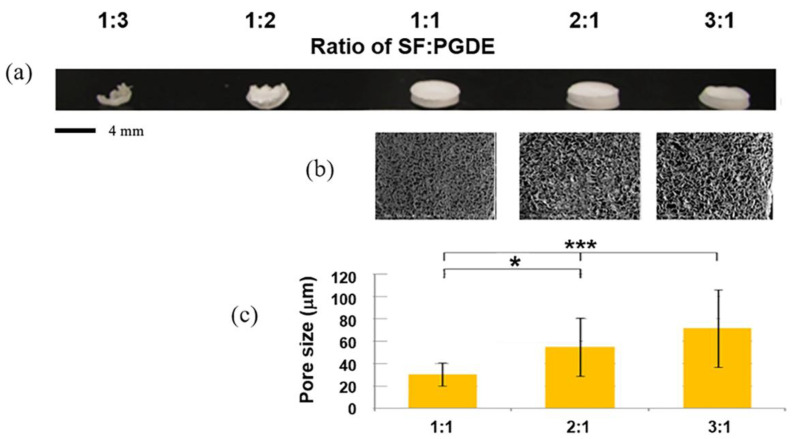
Examination of the conditions for the preparation of silk fibroin sponge using 5 *w/v*% silk fibroin aqueous solution and PGDE as a porogen. The ratios of silk fibroin/PGDE were changed. (**a**) Overviews, (**b**) SEM pictures, and (**c**) pore sizes of silk fibroin sponges. Finally, the preparation conditions for silk fibroin sponges are concluded to be a 5% aqueous solution and a ratio of SF:PGDE of 1:1. * *p* < 0.05, *** *p* < 0.0001.

As an example, the conditions for the preparation of silk sponges using PGDE as a porogen suitable for coating silk vascular grafts were investigated [109]. SF aqueous solutions with concentrations of 1 *w/v*% and 5 *w/v*% were prepared, and sponges were made by varying the ratios of PGDE to SF. The samples made with 1 *w/v*% SF solution did not form sponges but instead became hard, solid masses, making them unsuitable for the production of porous artificial vascular grafts. The results for the 5 *w/v*% SF aqueous solution are shown in Figure 10. The samples with ratios of SF:PGDE of 1:3 and 1:2 also became hard, solid masses. The measured pore sizes tended to become larger with decreasing ratios of SF:PGDE from 1:1 to 3:1, and the ratio of 1:1 resulted in a suitable pore size favorable for endothelial cell migration [121]. Therefore, the preparation conditions for SF sponges are concluded to be a 5% aqueous solution and a ratio of SF:PGDE of 1:1.

In order to study the structure and dynamics of SF sponge in the dry and hydrated states, solid-state NMR can be used effectively. The two leading analytical methods capable of structural analysis at the atomic level are X-ray analysis and NMR. Since SF consists of both crystalline and amorphous domains, it is difficult to obtain information from the amorphous domains using X-ray analysis. Therefore, solid-state NMR is currently regarded as the most suitable method for evaluating vascular grafts, as it can provide structural information from both crystalline and amorphous domains. Furthermore, since vascular grafts are used in hydrated environments, characterizing their structure and dynamics in the hydrated state is essential. Solid-state NMR is also well-suited for this purpose. Three distinct ^13^C NMR methodologies—refocused Insensitive Nuclei Enhanced by Polarization Transfer (r-INEPT), CP/MAS, and dipolar decoupled/magic angle spinning (DD/MAS)—were employed to investigate the structural and dynamic characteristics of hydrated SF sponges [122]. The r-INEPT technique, originally designed for solution-state NMR, is particularly responsive to rapidly moving molecular segments in hydrated SF. Conversely, CP/MAS NMR relies on a magnetization transfer from abundant ^1^H nuclei to less abundant ^13^C nuclei, making it suitable for detecting rigid molecular regions. However, it struggles to capture ^13^C signals from segments undergoing dynamic fluctuations around 10^8^ Hz, thus limiting its sensitivity to slowly moving components. DD/MAS NMR, on the other hand, can detect both fast and slow molecular motions and is capable of quantifying conformational distributions within the hydrated SF matrix. Together, these three techniques offer complementary insights into the molecular architecture and mobility within hydrated SF sponges. Furthermore, selective ^13^C isotope labeling of specific amino acid residues—[3-^13^C]Ser, [3-^13^C]Tyr, and [3-^13^C]Ala—enhances the resolution and specificity of the analysis. Ser is predominantly found in crystalline regions, Tyr in amorphous regions, and Ala in both. This labeling strategy enables site-specific conformational analysis in both ordered and disordered regions under hydrated conditions.

**Figure 11:**
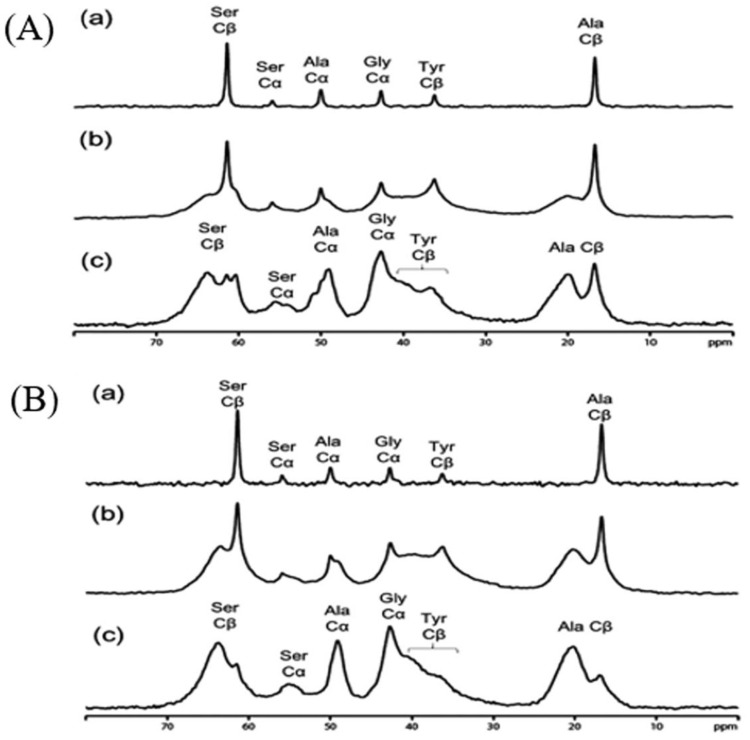
Expanded ^13^C solid-state NMR spectra of hydrated [3-^13^C]Ser-, [3-^13^C]Tyr-, and [3-^13^C]Ala- silk fibroin sponges prepared using (**A**) Glyc and (**B**) PGDE (SF/Glyc and SF/PGDE ratios are 1:1 *w/w*) as the porogens together with spectral assignments. (a) ^13^C r-INEPT, (b) ^13^C DD/MAS, and (c) ^13^C CP/MAS NMR spectra. Reprinted with permission from [122].

**Figure 12:**
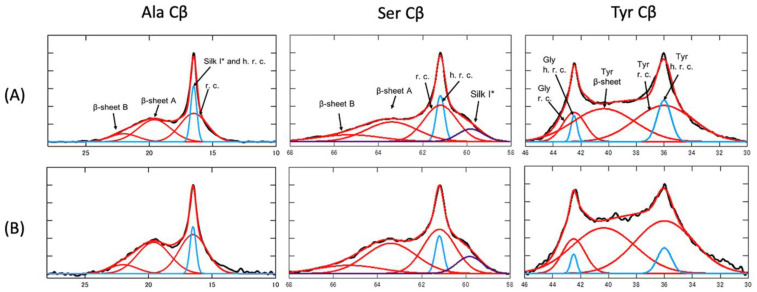
Observed and deconvoluted ^13^C DD/MAS NMR peaks of Ala Cβ, Ser Cβ, and Tyr Cβ carbons of hydrated [3-^13^C]Ala-, [3-^13^C]Ser-, and [3-^13^C]Tyr- silk fibroin sponges prepared using (**A**) Glyc and (**B**) PGDE as porogens (SF/PGDE ratio is 1:1 *w/w*). Here, r.c.: random coil and h.r.c.: hydrated random coil. The fractions are (**A**) Ala Cβ: β-sheet B 11.0, β-sheet A 35.0, r.c. 37.6, Silk I* + h.r.c. 16.4, (**B**) Ala Cβ: β-sheet B 14.0, β-sheet A 44.7, r.c. 32.7, Silk I* + h.r.c. 8.6, (**A**) Ser Cβ: β-sheet B 12.9, β-sheet A 30.0, r.c. 35.9, h.r.c. 11.5, Silk I* 9.7, (**B**) Ser Cβ: β-sheet B 14.3, β-sheet A 42.1, r.c. 30.9, h.r.c. 7.6, Silk I* 5.1, (**A**) Tyr Cβ: β-sheet 43.2, r.c. 44.5, h.r.c. 12.3, (**B**) Tyr Cβ: β-sheet 53.8, r.c. 40.6, h.r.c. 5.6, (**A**) Gly Cα:un-hydrated 80.5, hydrated 19.5, (**B**) Gly Cα:un-hydrated 78.2, hydrated 21.8. Reprinted with permission from [122].

Figure 11 displays expanded ^13^C solid-state NMR spectra of SF sponges labeled with [3-^13^C]Ser, [3-^13^C]Tyr, and [3-^13^C]Ala, prepared using either Glyc or PGDE as porogens at a 1:1 weight ratio. The spectra include (a) r-INEPT, (b) DD/MAS, and (c) CP/MAS data, along with peak assignments [122]. Figure 12 presents the deconvoluted DD/MAS spectra used to estimate conformational fractions. The r-INEPT spectra, which reflect mobile random coil regions, show minimal differences between the two sponge types. In contrast, both DD/MAS and CP/MAS spectra reveal notable variations. The proportion of β-sheet structures (A + B), as determined from the Cβ signals of Ser and Ala, is over 10% higher in PGDE-SF sponges compared to Glyc-SF sponges. A similar trend is observed for the Tyr Cβ peak. Mechanical testing of the hydrated SF sponges (1:1 SF/porogen ratio) was conducted immediately after surface water removal. The breaking strength values were 0.18 (±0.00) MPa for Glyc-SF and 0.29 (±0.00) MPa for PGDE-SF. Meanwhile, elongation at break was 65 (±2) and 52 (±1) for Glyc-SF and PGDE-SF, respectively. These mechanical differences are closely linked to the higher β-sheet content in PGDE-SF sponges.

### 5.3. Preparation of Silk Knitted Small-Diameter Vascular Graft Coated with Silk Fibroin Sponge

**Figure 13:**
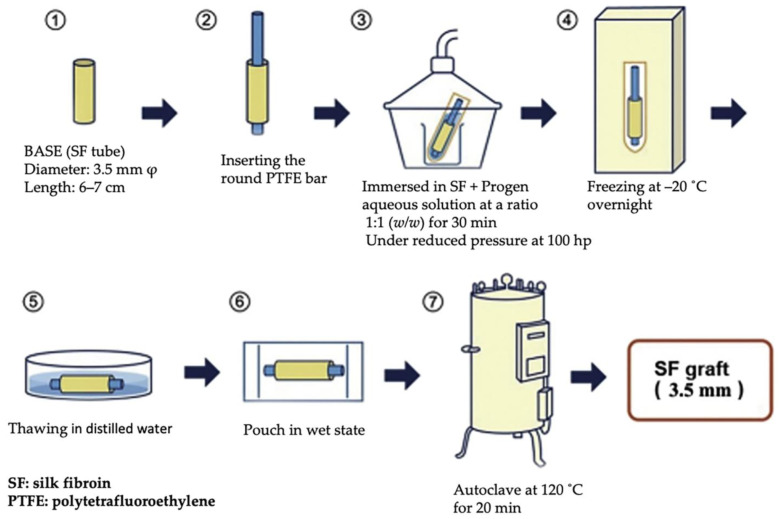
Preparation process of silk fibroin vascular graft with 3.5 mm diameter coated with silk fibroin sponge. Reprinted with permission from [112]. Copyright 2018 SAGE.

As previously described, the looped configuration characteristic of knitted textiles imparts superior stretchability, resilience, and conformability. The porous architecture intrinsic to these fabrics ensures excellent air and moisture permeability, as well as high porosity. These features are particularly beneficial when knitted materials are employed as artificial vascular grafts, as they support the efficient exchange of substances between the interior and exterior of blood vessels and encourage cellular infiltration. Compared to grafts produced via alternative fabrication techniques, knitted textiles with larger pore sizes offer improved blood flow dynamics [123]. Nevertheless, knitted structures tend to exhibit relatively low tensile strength within the plane of the fabric, and tailoring directional mechanical properties through knitting remains a significant challenge [124]. To address these limitations, applying an appropriate coating to the silk-based vascular graft is crucial. The fabrication steps for the SF vascular graft are illustrated in Figure 13 [112].

1. The foundation of the vascular graft is an SF tube produced using a double-raschel knitting technique (also known as double tricot), crafted from *B. mori* silk threads via a computer-controlled knitting machine. Before use, the silk undergoes a degumming process to eliminate residual sericin.

2. A PTFE rod is inserted into the knitted SF tube to maintain its shape during coating.

The outer surface of the tube is coated more effectively than the inner surface. However, both the entire outer and inner surfaces (which are coated through the gaps in the fiber assembly) are uniformly coated in their respective areas.

**Figure 14:**
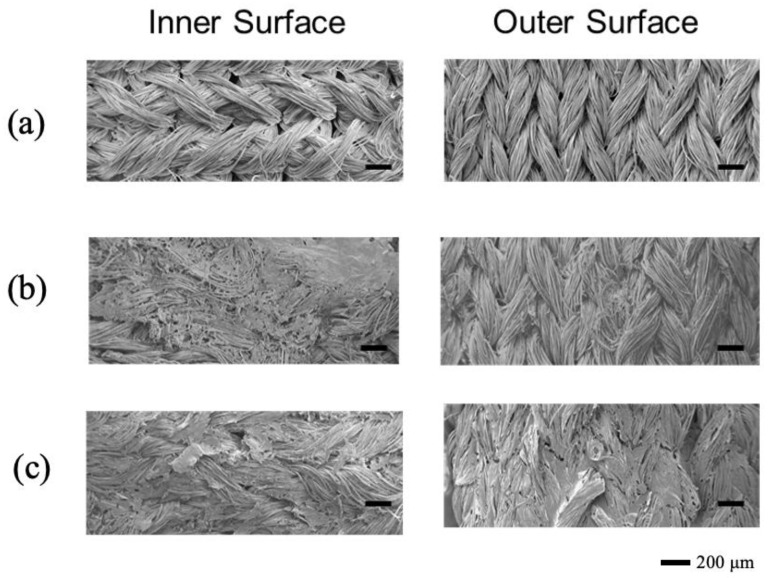
SEM pictures of the inner and outer surfaces of silk fibroin vascular grafts (**a**) before coating and (**b**,**c**) after coating with silk fibroin sponges. The porogens are (**b**) Glyc and (**c**) PGDE. The SF/porogen ratio is 1:1 *w/w*. These images confirm that the graft surfaces were uniformly and tightly coated with silk sponges. Reprinted with permission from [112]. Copyright 2018 SAGE.

3. The SF tube, now fitted with the rod, is submerged in a cylindrical container filled with an aqueous mixture of SF and a porogen—such as Glyc or PGDE—typically in a 1:1 weight ratio. The container is placed in a vacuum desiccator (100 hPa) until all air bubbles disappear from the surface, ensuring thorough coating. During the treatment process, the silk artificial blood vessel is immersed in the aqueous mixture, which remains sufficiently filled until the process is complete. Therefore, there is no change in the concentration of SF.

4. After this step, the coated SF graft is frozen at −20 °C and left overnight.

5. The frozen graft is then soaked in distilled water for three days to completely remove the porogen material.

6. Once cleaned, the graft is stored in a sealed pouch filled with distilled water.

7. For sterilization, the pouch containing the SF graft is autoclaved at 120 °C for 20 min, leveraging the high thermal resistance of silk fibroin. The sterilized graft remains in distilled water until it is ready for implantation in animal studies. Figure 14 displays SEM pictures of the inner and outer surfaces of SF vascular grafts before and after sponge coating using the two different porogens. These images confirm that the graft surfaces were uniformly and tightly coated with silk sponges.

### 5.4. Results of Implanted Small-Diameter Silk Vascular Graft in Rat

**Figure 15:**
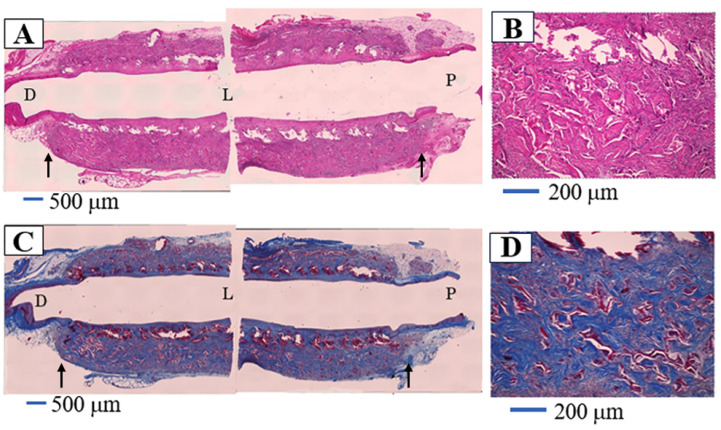
Pictures of (**A**) H&E staining and (**C**) MTC staining of longitudinal histological sections of the silk fibroin vascular graft coated with silk fibroin sponge using PGDE as a porogen, 4 weeks after transplantation into a rat. Here, H&E staining is a commonly used technique in histology to visualize tissue structure. MTC staining is used to differentiate between muscle, collagen, and other tissue components. Black arrows are the anastomosis area of the native aorta. D, L, and P in the pictures describe distal (**D**), proximal (P), and inner lumen (L) of the silk fibroin graft, respectively. The expanded pictures of (**A**) and (**C**) are also shown as pictures (**B**) and (**D**), respectively. Reprinted with permission from [109].

**Figure 16:**
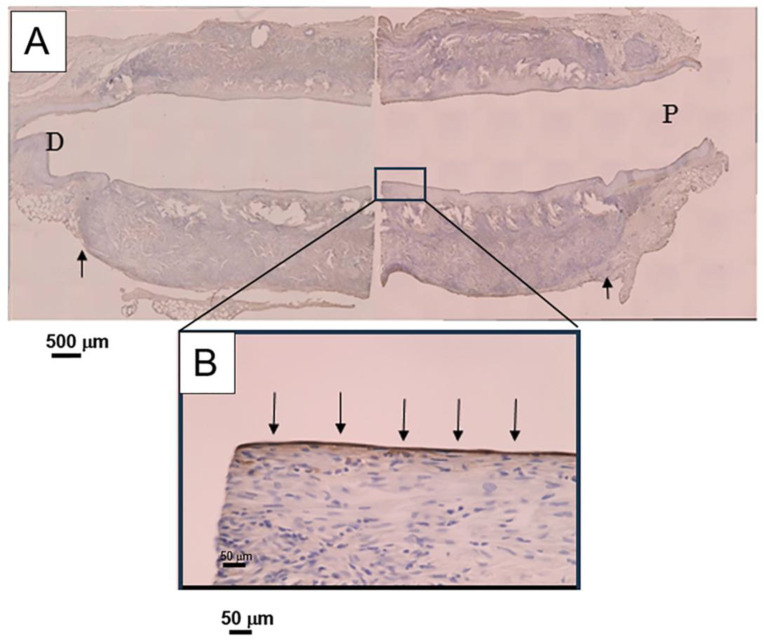
Pictures of the endothelial cell layer of the lumen surface of the silk fibroin vascular graft coated with silk fibroin sponge using PGDE as a porogen 4 weeks after transplantation into a rat. The graft is stained with anti-CD31 antibody. Here, staining with anti-CD31 antibody is a method used to detect blood vessels in tissue samples. CD31 is a protein found on the surface of endothelial cells, which line the inside of blood vessels. By using an antibody that specifically binds to CD31, researchers can visualize and study the blood vessel structures under a microscope. The general image of the longitudinal section (**A**) and the high magnification image of the middle portion (**B**) are shown. P and D on the figure each mean proximal and distal. Arrows on the A show anastomosis sites, and on the B, they show the lining of the endothelial cell layer. Reprinted with permission from [109].

SF grafts, measuring 1.5 mm in internal diameter and 1 cm in length, were coated with a silk sponge crosslinked using PGDE as a porogen and implanted via end-to-end anastomosis (surgical joining technique of a silk vascular graft to a native blood vessel to restore blood flow) into the abdominal aorta of rats. All surgical procedures followed previously established protocols [106,107]. During the implantation phase, no significant blood leakage was detected from any of the SF grafts. Figure 15A,C illustrate longitudinal histological sections of the porous SF vascular grafts stained with Hematoxylin and Eosin (H&E) and Masson’s Trichrome (MTC), respectively, after a 4-week implantation period [109]. Here, H&E staining is a commonly used technique in histology to visualize tissue structure. MTC staining is used to differentiate between muscle, collagen, and other tissue components. The graft lumen and the junctions with the native abdominal artery appeared smooth, with no evidence of thrombus formation or intimal thickening. The implanted SF grafts were infiltrated with well-organized native collagen layers. The silk sponge component had largely degraded, losing its original structure and becoming densely populated with infiltrating cells. Vascularization within the sponge matrix was also evident, as depicted in Figure 15B. In contrast to SF, PTFE has been widely reported as biologically stable and resistant to degradation [125]. A prior study analyzed 79 explanted ePTFE grafts using techniques such as contact angle measurement, electron spectroscopy for chemical analysis (ESCA/XPS), FTIR, and DSC [126]. These results, compared with virgin and washed PTFE samples, showed no signs of chemical degradation even after 6.5 years of implantation [127]. Conversely, SF materials—including fibers, films, powders, and regenerated forms—have been shown to degrade in vivo through enzymatic hydrolysis by proteases and chymotrypsin, often accompanied by inflammatory responses [53,126,128,129]. In this study, acute inflammation was evident, marked by macrophage infiltration and the presence of multinucleated giant cells. The immune activity of blood cells significantly contributed to the breakdown of the SF sponge. MTC staining revealed that the SF sponge promoted collagen deposition along the vascular wall, aiding in the reconstruction of the extracellular matrix (Figure 15C,D). Figure 15D shows the sponge completely degraded and replaced by collagen-rich tissue and infiltrating cells. After four weeks, remnants of the sponge appeared as red shadows within the native tissue, indicating active tissue migration and matrix reconstruction within the porous SF scaffold. Immunostaining of the luminal surface of the SF graft with Cluster of Differentiation 31 (CD31) antibody revealed the presence of an endothelial cell layer, as illustrated in Figure 16A [109]. Here, staining with anti-CD31 antibody is a method used to detect blood vessels in tissue samples. CD31 is a protein found on the surface of endothelial cells, which line the inside of blood vessels. By using an antibody that specifically binds to CD31, researchers can visualize and study the blood vessel structures under a microscope. A magnified view in Figure 16B demonstrates that endothelial cells had successfully colonized the entire inner surface of the graft, including its central region, by the fourth week post-implantation. Earlier research reported that endothelial coverage on the SF graft lumen reached approximately 30–40% at two weeks and approached full coverage (nearly 100%) by eight weeks [101]. Given the excellent patency and substantial tissue integration observed in the rat model within just four weeks, these porous SF grafts exhibit strong promise as candidates for vascular reconstruction applications.

### 5.5. Results of Implanted Small-Diameter Silk Vascular Graft in Dog

**Figure 17:**
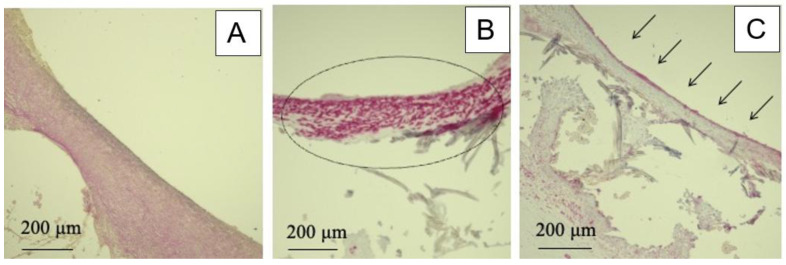
Tissue images at 3 months after transplantation of the silk fibroin vascular graft coated with silk fibroin sponge using Glyc as a porogen into the femoral artery of beagle dogs. (**A**) EVG staining, (**B**) α-SMA staining, and (**C**) CD31 staining are shown. Here, EVG (Elastica van Gieson) staining is a histological technique used to highlight elastic fibers in tissue sections. α-SMA is a protein commonly found in smooth muscle cells and myofibroblasts. Elastic fibers, smooth muscle cells (indicated by black circles), and endothelial cells (indicated by arrows) can be observed in the intimal layer and on the surface of the graft. Reprinted with permission from [114]. Copyright 2021 Elsevier.

To date, most experiments using small-diameter vascular grafts made of SF have been conducted on the abdominal aorta of rats, and it has been demonstrated that SF vascular grafts exhibit high patency rates and remodeling capabilities in vivo. [82,83,106,108,109,110,112]. However, these studies mainly focused on the abdominal aorta of rats, and evaluating SF vascular grafts in larger animal models has been a challenge for clinical application in human medicine [24,25,26]. In a study examining the long-term patency of small-diameter grafts in the carotid artery of dogs, no significant difference was observed between SF grafts and existing ePTFE grafts. However, endothelialization of the central part of the graft was incomplete [107]. One reason for the low patency of SF-coated grafts in dogs is the delayed biodegradation and remodeling of SF grafts. To address this issue, researchers replaced alcohol-based insolubilization with a mixture of SF and Glyc (used as a pore source) to develop a more degradable coating [123]. The resulting SF grafts were more flexible and had superior remodeling capabilities compared to conventional SF grafts insolubilized with alcohol [112]. Subsequently, SF grafts coated with an amorphous SF sponge were transplanted into the femoral artery of dogs to investigate the patency and remodeling capabilities of these new small-diameter SF vascular grafts in a large animal model. SF grafts (4 cm in length, 3.5 mm in inner diameter) were transplanted into both femoral arteries of six dogs [114]. At 3 months, 5 months, and 1 year after transplantation, the grafts were removed under general anesthesia following the same procedure as the initial surgery. After removing the grafts, standard staining methods such as H&E, MTC, Elastica van Gieson (EVG), and immunohistochemical staining (α-Smooth Muscle Actin (SMA) and CD31) were applied. In the in vivo experiments, no side effects such as hind limb paralysis or hematoma were observed. Here, EVG (Elastica van Gieson) staining is a histological technique used to highlight elastic fibers in tissue sections. The α-SMA is a protein commonly found in smooth muscle cells and myofibroblasts. Furthermore, there was no change in the adhesion strength between the graft and surrounding tissue at 3 months, 5 months, and 1 year after transplantation. However, in one case, occlusion due to thrombosis occurred 4 weeks after transplantation. Histopathological analysis using H&E staining revealed a layered structure along the luminal surface of the graft. Notably, the thickness of this structure increased at 5 months and 1 year compared to 3 months after transplantation, but no luminal stenosis was observed. The layered structure was mainly composed of smooth muscle cells, elastic fibers, and collagen fibers, with no significant differences in the components observed during each transplantation period. As shown in Figure 17 [114], at 3 months after transplantation, smooth muscle cells and elastic fibers were observed in the lumen of the central part of the graft, and the innermost surface was lined with endothelial cells. Notably, no granulomas, aneurysms, or calcifications were observed in the remodeled grafts. The patency rate of the transplanted vascular grafts exceeded 80% after transplantation. Remodeling into autologous tissue was similar to that observed in rat transplants at 3 months. As the post-transplantation period increased, the thickness of the intima gradually increased without narrowing the lumen. Endothelial cells are believed to play an important role in preventing luminal stenosis due to blood coagulation and excessive proliferation of smooth muscle cells [130]. Importantly, this study showed that even at 1 year after transplantation, no aneurysm rupture or calcification occurred, and high patency was maintained. Evaluating transplants in larger animals was essential for the clinical application of SF artificial vascular grafts. In this study, small-diameter SF vascular grafts transplanted into the femoral artery of dogs demonstrated both high patency and remodeling capabilities. SF grafts were suggested to be promising as clinically applicable artificial vascular grafts with a small diameter (<6 mm).

## 6. Small-Diameter Silk Fibroin Vascular Grafts with Modified Silk Fibroin

To expand silk-based applications like vascular grafts, chemical and transgenic strategies for structural modification are essential [131]. Hemocompatibility depends on luminal surface traits. Sulfonation and heparin conjugation have been studied to enhance endothelial adhesion and reduce platelet attachment and clotting [23].

### 6.1. Chemical Modification of Silk Fibroin

Tamada [132] sulfated SF using chlorosulfonic acid and confirmed sulfate attachment to Ser and Tyr via IR and Raman analysis. Liu et al. [133] created sulfated SF nanofibers (S-silk) by electrospinning, showing enhanced blood compatibility and vascular cell adhesion with increased marker expression. Compared to regular SF scaffolds, S-silk had superior anticoagulant properties. In a follow-up study, rat aortic endothelial cells (ECs) cultured under pulsatile flow in a bioreactor showed improved endothelial function and thromboresistance [134]. Furthermore, a composite scaffold was engineered by integrating sulfated SF sponges into a knitted silk framework [133,135], which demonstrated superior hemocompatibility, facilitated EC adhesion and growth, and preserved cellular activity. Heparin is widely recognized in vascular tissue engineering for its anticoagulant efficacy and its role in improving hemocompatibility. Heparin-functionalized grafts have shown resistance to thrombosis and enhanced stability and release profiles of the vascular endothelial growth factor (VEGF) [136,137,138,139]. Zhu et al. [92,93] introduced a mild freeze-drying approach to produce porous 3D SF scaffolds. In vitro experiments revealed that these scaffolds promoted cell infiltration, suppressed abnormal smooth muscle cell (SMC) proliferation, and improved blood compatibility. When implanted subcutaneously in rats, the heparin-loaded SF scaffolds significantly stimulated new blood vessel formation. Similarly, Seib et al. [139] demonstrated that heparinized SF materials could serve dual functions—enhancing hemocompatibility and enabling controlled growth factor delivery. Saitow et al. [140] created heparin-infused SF films by drying a silk–heparin mixture on glass substrates for 24–48 h. Culturing human SMCs on these films revealed that heparin promoted elastic fiber organization. Electrospun SF nanofibers were also modified with heparin via post-plasma grafting and blending electrospinning techniques [141], resulting in improved cell spreading and proliferation of fibroblasts and ECs compared to unmodified SF scaffolds. In vivo studies confirmed the biocompatibility of these heparin-modified constructs. Zamani et al. [142] reported that scaffolds with covalently bonded heparin maintained long-term antithrombotic activity in vitro. To assess the impact of Arg-Glu-Asp-Val (REDV) peptide functionalization, cell culture studies using HUVECs and human aortic smooth muscle cells (HASMCs) were conducted on SF and SF + REDV films [143]. The results suggested that REDV-modified SF promoted HUVEC proliferation while suppressing HASMC growth, indicating its potential suitability for coating small-diameter silk-based vascular grafts.

### 6.2. Transgenic Silk Fibroin

To engineer SF with properties suitable for biomedical applications [144], researchers have developed a technique utilizing the piggyBac transposon system for generating transgenic (TG) silkworms [145]. This approach enables the integration of specific DNA sequences, thereby improving the functional characteristics of silk fibroin. This method offers several advantages: (1) TG silkworms are easy to handle and thrive under artificial breeding conditions. (2) Adult moths are flightless and incapable of surviving in natural environments. (3) The production of recombinant proteins can be visually tracked by attaching Green Fluorescent Protein (GFP) markers [146,147]. SF from B. mori comprises two main components: a heavy chain (H-chain; molecular weight ~350 kDa) and a light chain (L-chain; ~25 kDa) [33]. These chains, along with the P25 protein, are synthesized in the silk gland cells and secreted into the gland. The cocoon typically contains these chains in a 1:1 ratio. When a new gene is inserted downstream of the fibroin gene, it is expressed as a novel protein within the silk gland. Saotome et al. [148] highlighted the incorporation of two bioactive sequences—Arg-Gly-Asp (RGD) from fibronectin and VEGF—to enhance endothelial cell adhesion and reduce thrombogenicity. VEGF promotes endothelial cell proliferation and inhibits platelet adhesion by preventing cell rolling. Fluorescence microscopy images show HUVEC attachment to three SF variants. Both VEGF-modified SF and RGD-modified SF exhibited significantly improved HUVEC adhesion and proliferation compared to wild-type SF. Platelet binding was more pronounced in RGD SF and WT SF, while VEGF SF showed markedly reduced platelet adhesion. Thus, RGD SF facilitated strong HUVEC and platelet attachment, whereas VEGF SF supported robust HUVEC adhesion but minimized platelet interaction. Consequently, VEGF-enhanced SF materials demonstrated superior patency and tissue integration, promoting neovascularization. Among the tested sequences, VEGF and REDV were most effective in supporting endothelialization and tissue penetration in small-diameter SF-based vascular grafts [116].

## 7. Small-Diameter Silk Fibroin Vascular Grafts with Silk Fibroin Composite Materials

An alternative approach to functional integration is physically mixing silk proteins with other substances to form stable composites [149]. These can be structured via electrospinning or copolymerization. Various SF-based composites have been developed, including blends with natural and synthetic polymers and modifications like heparin blending or grafting [150,151,152,153,154,155].

### 7.1. Silk Fibroin Vascular Grafts with Silk-Biodegradable Polyurethane Sponge

**Figure 18:**
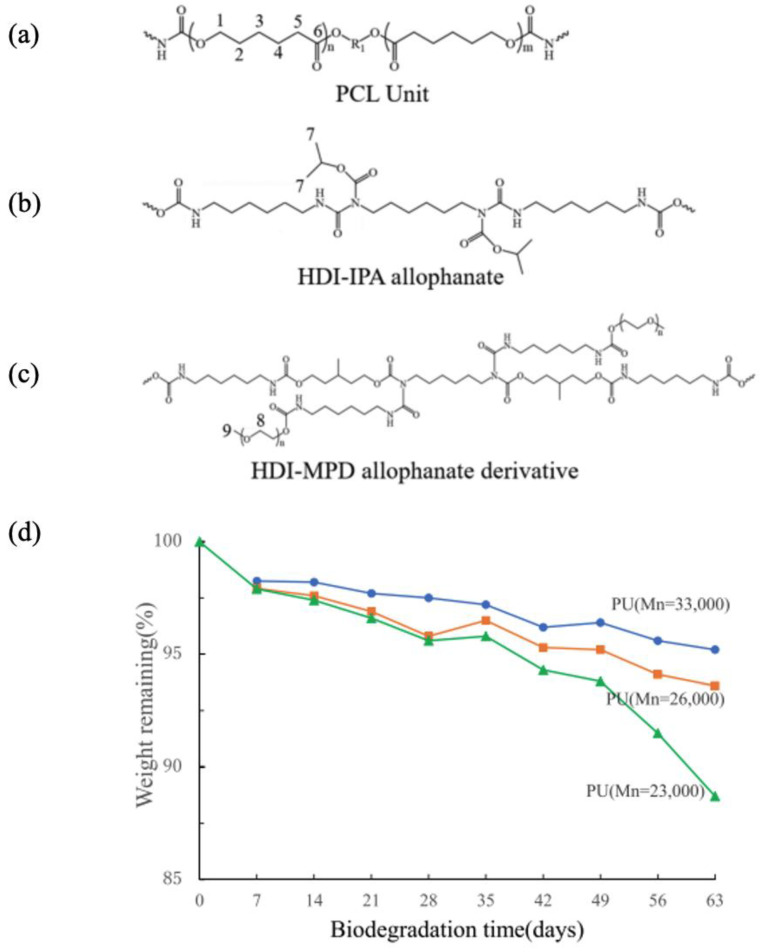
Structural formula of (**a**) PCL unit, (**b**) HDI-IPA allophanate, and (**c**) HDI-MPD allophanate derivative, which are the components of biodegradable PU. (**d**) Time dependences of degradation of three PU samples with different molecular weights. Reprinted with permission from [159]. Copyright 2021 Elsevier.

The rigidity of SF fiber grafts coated with SF has been noted as a concern, potentially affecting transplant outcomes due to issues like thrombus formation and compliance mismatch [53,127,128]. To address this, a more flexible and biodegradable SF graft has been proposed. This graft is fabricated by applying a biodegradable PU-SF composite sponge onto a small-diameter, double-raschel knitted SF vascular scaffold [115,156,157,158,159]. The biodegradable PU was synthesized using polycaprolactone (PCL) segments, hexamethylene diisocyanate (HDI)-2-propanol (IPA) allophanate, HDI-3-methyl-1,5-pentanediol (MPD) allophanate derivatives, and other constituents [159]. Figure 18 illustrates the chemical structures of (a) the PCL unit, (b) HDI-IPA allophanate, and (c) HDI-MPD allophanate derivative, with PCL diol serving as the principal component. A degradation study was conducted on three PU samples with varying molecular weights using phosphate-buffered saline, and the outcomes are presented in Figure 18d. The percentage of remaining weight in the PU films declined progressively over time, with the most significant reduction observed at 63 days in the sample with the lowest molecular weight (Mn = 23,000). Consequently, this particular PU formulation was selected for subsequent coating applications on the SF graft [160,161,162]. To evaluate in vivo biodegradability, the water-dispersible PU-SF composite sponges were implanted into the dorsal subcutaneous tissue of rats.

### 7.2. Structure of Silk Fibroin–Polyurethane Sponge

**Figure 19:**
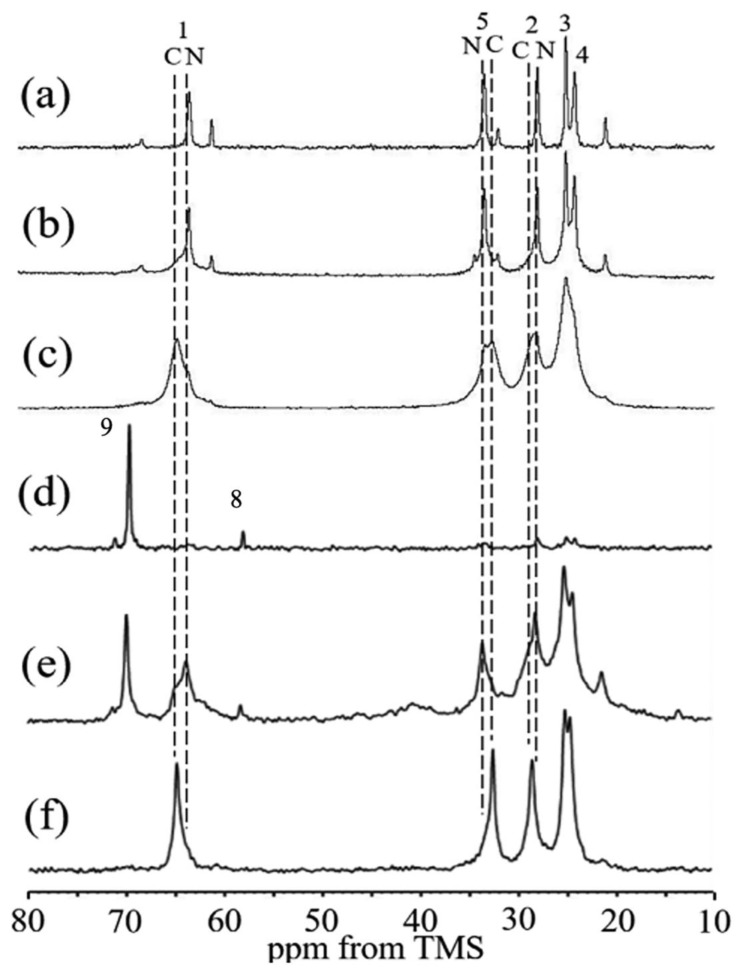
Expanded (**a**) ^13^C r-INEPT, (**b**) ^13^C DD/MAS, and (**c**) ^13^C CP/MAS NMR spectra (10–80 ppm) of the hydrated PCL diol and expanded (**d**) ^13^C r-INEPT, (**e**) ^13^C DD/MAS, and (**f**) ^13^C CP/MAS NMR spectra (10–80 ppm) of PU. C and N mean crystalline and non-crystalline regions of PCL diol, respectively. The number of each carbon in PU is described in Figure 17A–C. Reprinted with permission from [159]. Copyright 2021 Elsevier.

**Figure 20:**
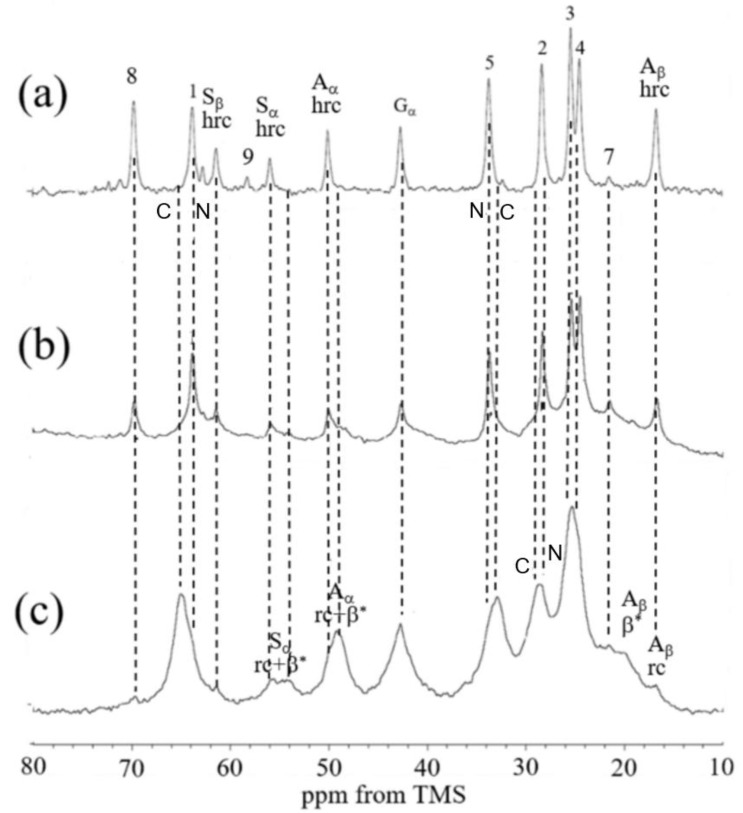
Expanded (**a**) ^13^C r-INEPT, (**b**) ^13^C DD/MAS, and (**c**) ^13^C CP/MAS NMR spectra (10–80 ppm) of the SF-PU sponge in the hydrated state. C and N mean crystalline and non-crystalline regions of PCL diol, respectively. β*: antiparallel β-sheet, rc: random coil, and hrc: hydrated random coil. The number of each carbon indicates the PU peaks noted in Figure 17A–C. Reprinted with permission from [115]. Copyright 2021 MDPI.

To begin with, the molecular structure of the biodegradable PU was analyzed in its hydrated form using ^13^C solid-state NMR spectroscopy, and the results were compared with those of PCL diol, the primary component of the PU. Figure 19 presents the expanded spectral region (10–80 ppm) of the ^13^C solid-state NMR data, including the following: (a) ^13^C r-INEPT, (b) ^13^C DD/MAS, and (c) ^13^C CP/MAS spectra for PCL diol, as well as (d) ^13^C r-INEPT, (e) ^13^C DD/MAS, and (f) ^13^C CP/MAS spectra for the hydrated biodegradable PU [159]. The observed doublet peaks were attributed to carbon atoms 1, 2, and 5 located in both crystalline (C) and amorphous (N) domains of the PCL diol [163,164] or to the corresponding carbons in the PCL segments of the PU. The chemical shift differences between the C and N peaks are explained by the ^13^C γ-gauche effect associated with the alkyl chains [164,165]. Notably, in spectrum (f), the crystalline peaks are significantly more intense than the amorphous ones, whereas in spectrum (c), the difference is less pronounced. In the DD/MAS spectra, the amorphous peaks dominate in spectrum (b), while both crystalline and amorphous peaks are clearly visible in spectrum (e). In the r-INEPT spectra, only amorphous peaks appear in spectrum (a), and only faint methylene signals from the PCL units are detected in spectrum (d). These findings suggest that the mobility of PCL diol chains within the PU matrix is restricted, even in the amorphous regions, under hydrated conditions. Additionally, sharp signals corresponding to carbons 8 and 9 in the terminal groups of the HDI-MPD allophanate side chains were observed, indicating that these segments retain mobility in the hydrated PU. Subsequently, the structure of the SF-biodegradable PU sponge was investigated using ^13^C solid-state NMR under hydrated conditions. The preparation procedure for the SF-PU sponge was as follows: An aqueous SF solution was mixed with Glyc in a 1:1 weight ratio (SF-Glyc) [112], then combined with a water-dispersed biodegradable PU solution in a 1:1 ratio (SF-PU). The resulting SF-PU-Glyc mixture was frozen at −20 °C overnight. After thawing in distilled water, Glyc was removed, and the sponge was sealed in a pouch. The final SF-PU sponge, immersed in water, was sterilized by autoclaving at 120 °C for 20 min. Figure 20 presents the expanded spectral regions (10–80 ppm) for (a) ^13^C r-INEPT, (b) ^13^C DD/MAS, and (c) ^13^C CP/MAS NMR analyses of the hydrated SF-biodegradable PU sponge [115]. In spectrum (c), the ^13^C CP/MAS NMR profile of the hydrated PU-SF sponge reveals distinct signals corresponding to both SF and PCL components. Notably, the Ala Cα, Ala Cβ, and Ser Cα peaks indicate the presence of both random coil and β-sheet structures in SF. Additionally, methylene carbon signals (positions 1, 2, and 5) from the PCL segments of PU are clearly visible. In the ^13^C DD/MAS spectrum, β-sheet peak intensities are reduced, while signals from hydrated random coil conformations are enhanced in the SF region. Similarly, the PU spectrum shows a marked decrease in the intensity of C peaks. The ^13^C r-INEPT spectrum exclusively displays hydrated random coil signals for SF, and interestingly, the N peaks of PCL carbons are distinctly observed, contrasting with the r-INEPT spectrum in Figure 19a. Sharp signals from carbons 8 and 9, located in the terminal groups of HDI-MPD allophanate side chains, are also detected. These observations suggest that PU is more uniformly blended with SF in the composite sponge and exhibits greater flexibility in aqueous environments. Consequently, water-dispersible PU-SF sponges appear to be more suitable for coating knitted SF grafts.

SF-PU composite biomaterials have been extensively explored for cardiovascular applications [87,115,156,157,158,159,166]. However, conventional PU used in these composites is typically non-biodegradable, posing challenges for its use as a coating material alongside biodegradable SF [87,157,166]. In small-diameter SF vascular grafts, non-degradable coatings hinder remodeling post-implantation, potentially leading to occlusion due to thrombus formation and intimal hyperplasia [114]. In contrast, the SF sponge developed in this study, incorporating water-dispersible degradable PU [115,159], demonstrated early biodegradation after implantation, making it a promising coating material for small-diameter vascular grafts.

During graft implantation, two types of SF grafts were used: one coated solely with a PU sponge and the other with a PU-SF composite sponge [115]. All 24 procedures were successfully completed without major complications such as excessive bleeding. Upon restoration of blood flow, no bleeding was observed from the PU-coated grafts, with only minor leakage at the anastomosis site. In contrast, the PU-SF-coated grafts exhibited mild bleeding from both the graft and the anastomosis site, which was easily controlled by brief compression with a cotton swab. Both graft types demonstrated similar handling and suturing characteristics. All rats survived until the designated graft retrieval times (2 and 4 weeks). At 2 and 4 weeks post-implantation, five out of six rats with PU-coated SF grafts experienced occlusion, whereas all rats with PU-SF-coated grafts maintained graft patency. The grafts were surrounded by a thin layer of adipose and connective tissue but could be easily separated from adjacent structures.

**Figure 21:**
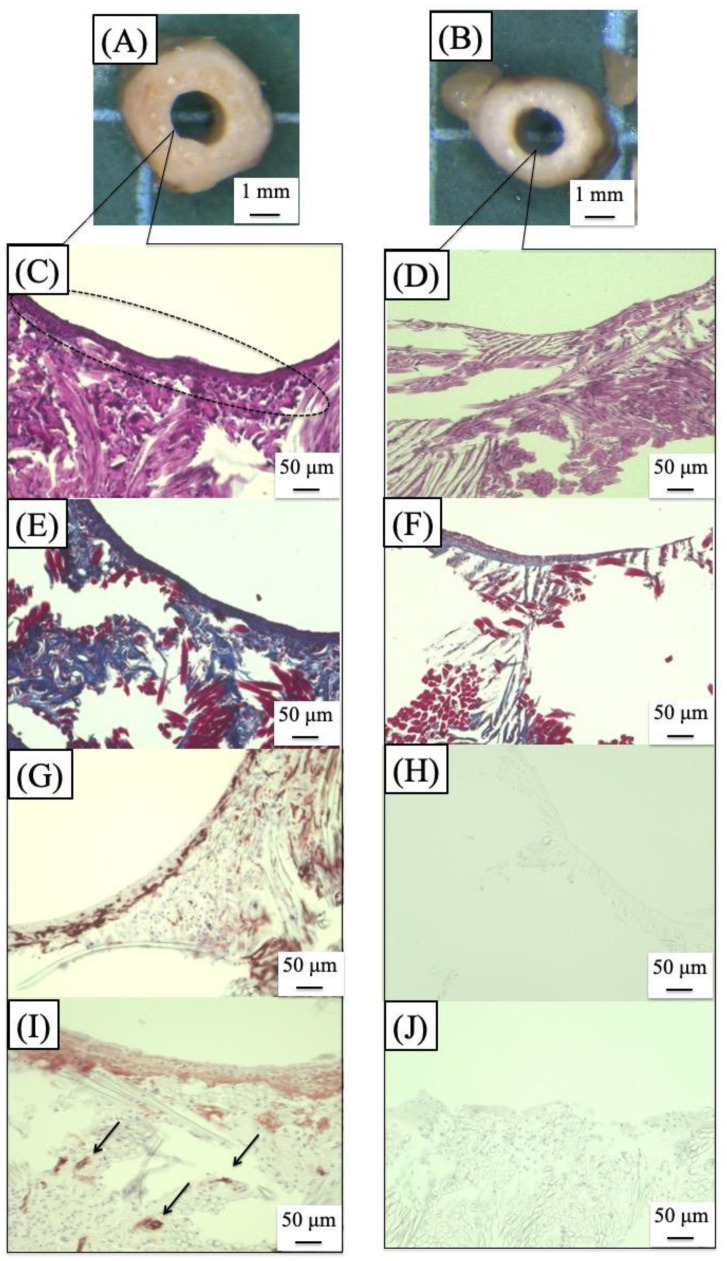
Photographs of the microscopic findings of the PU-SF-coated (**A**) and PU-coated grafts (**B**) at 2 weeks after implantation. Histological cross-sectional images of the PU-SF-coated graft at 2 weeks after implantation: the PU-SF-coated graft after (**C**) H&E, (**E**) EVG, (**G**) α-SMA, and (**I**) CD31 staining. Histological cross-sectional images of the PU-coated graft at 2 weeks after implantation: the PU-coated graft after (**D**) H&E, (**F**) EVG, and (**H**) α-SMA staining, and (**J**) the PU-SF-coated graft after CD31 staining. The part enclosed by the dotted line shows the intima. The arrows indicate that vascular endothelial cells adhered to the inner surfaces of the grafts. Reprinted with permission from [115]. Copyright 2021 MDPI.

Histological analysis using H&E staining at 2 weeks revealed the presence of inflammatory cells—including lymphocytes, macrophages, and neutrophils—both around and within the PU-SF-coated SF grafts. These immune responses were especially pronounced on the external surfaces of the grafts (Figure 21C) [115]. In comparison, PU-coated SF grafts also showed inflammatory cells on their outer surfaces, similar to the PU-SF group, but no such cells were detected within the graft interiors (Figure 21D). MTC staining demonstrated that collagen fibers had infiltrated throughout the PU-SF-coated grafts, penetrating the interstitial spaces of the graft matrix (Figure 21E). Conversely, in the PU-coated grafts, collagen accumulation was limited to the outer surface, with minimal penetration into the graft interior (Figure 21F). Immunostaining for α-SMA revealed that smooth muscle cells were primarily localized along the outer layer and the luminal surface of the PU-SF-coated grafts (Figure 21G). However, in the PU-only coated grafts, smooth muscle cells were absent from the luminal surface (Figure 21H). CD31 staining performed two weeks after implantation did not show endothelial cell coverage on the luminal surfaces of either graft type. Nevertheless, in the PU-SF-coated grafts, several blood vessels were observed infiltrating the porous regions of the graft matrix (Figure 21I) [167].

**Figure 22:**
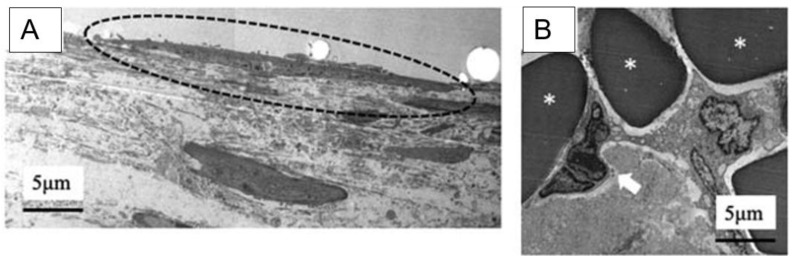
Transmission electron microscopy (TEM) images of SF-PU-coated SF grafts 4 weeks after transplantation into a rat. (**A**) The dotted area indicates endothelial cells attached to the inner surface of the graft. (**B**) The arrows show autologous tissue infiltrating the gaps between the silk fibers (asterisks). Reprinted with permission from [167]. Copyright 2024 Elsevier.

At four weeks post-implantation, partial endothelialization was observed on the luminal surface of the PU-SF-coated grafts, although complete coverage had not yet been achieved. In contrast, the PU-coated grafts still lacked endothelial cells on the inner surface, consistent with the two-week findings. To gain deeper insight into structural integration, transmission electron microscopy (TEM) was used to examine the PU-SF-coated grafts at four weeks. Host-derived cells were found to have migrated into the inter-fiber spaces of the SF scaffold (Figure 22B), and endothelial cells were confirmed to be attached to the inner surface of the artificial vessel (Figure 22A).

The PU-coated grafts did not exhibit any blood leakage during surgery or excessive inflammatory responses afterward, indicating acceptable biocompatibility. However, their post-implantation patency rate was low. Histological evaluation of the patent PU-coated grafts showed poor host cell infiltration, suggesting that the PU coating alone did not provide sufficient porosity. Previous studies using PU-based vascular grafts have shown that pore size significantly influences tissue integration and remodeling [168]. Therefore, further research is needed to determine whether adequate porosity can be achieved with PU-only coatings. In contrast, the PU-SF-coated SF grafts developed in this study demonstrated degradation of the coating within two weeks, allowing host cells to infiltrate the graft matrix. The presence of endothelial cells on the luminal surface at four weeks supports the conclusion that PU-SF-coated SF grafts are promising candidates for use as coatings in silk fibroin-based vascular grafts.

In addition to PU, other synthetic or natural polymers used in combination with silk fibroin for the fabrication of small-diameter vascular grafts include polycaprolactone (PCL), polylactic acid (PLA), thermoplastic polyurethane (TPU), poly(glycerol sebacate) (PGS), and poly(lactic-co-glycolic acid) (PLGA). Natural polymers include polysaccharides, collagen, fibrinogen, chitosan, elastin, and cellulose.

## 8. Conclusions and Future Prospects

The structural characteristics of SF before and after fiber formation were thoroughly investigated using solid-state NMR techniques. Building on these insights, the development of small-diameter SF-based vascular grafts is discussed, with emphasis on fabrication strategies. Particular focus is placed on knitted SF grafts enhanced with SF sponge coatings, whose molecular structures were examined in both dry and hydrated states using ^13^C solid-state NMR. The grafts’ biological performance was evaluated through implantation studies in rat and canine models. Additionally, this study explores chemically modified SF grafts, such as those incorporating silk–polyurethane composite sponges, highlighting their structural properties and in vivo outcomes.

However, these studies are still in their early stages. The SF composite grafts have been successfully implanted into small arteries, showing promising results in terms of cell compatibility, controlled biodegradability, and short-term patency. While these outcomes are highly encouraging, many challenges remain before clinical application can be realized. The ideal artificial vascular graft needs to satisfy several competing criteria. Mechanical properties need to be tuned to combine high strength with elasticity that closely matches the native vasculature. Targeting burst pressures well in excess of normal human physiological conditions (>1000 mmHg) and high tensile strength (>1 MPa) combined with appropriate compliance (10–20%/100 mmHg) [169]. However, the mechanical properties required for the in vivo use of small-diameter vascular grafts vary depending on age, sex, and health status, making the design of vascular morphology, structure, and thickness challenging [170]. For example, recent studies have shown that small-diameter vessels have a higher maximum strength than large-diameter vessels [171]. The size of vascular grafts also varies depending on the body part and individual. In addition, blood vessels expand with age and may rupture under hypertension. The thickness of the inner vascular layer and the likelihood of thrombus (blood clot) formation increase over time, and this process is more strongly influenced by lifestyle than by age or sex.

On the other hand, after implantation of the vascular grafts, key questions include whether the SF intimal layer can function as a biocompatible and durable interface over the long term, what the most effective strategies are for graft delivery and implantation, and whether it can be used in combination with large-vessel revascularization procedures. Furthermore, it remains unresolved whether auxiliary factors such as vascular endothelial growth factors are necessary to enhance long-term patency and whether the SF grafts can be designed into long-length structures suitable for long-term implantation. Addressing these issues is essential to transform the current “proof of potential” into a clinically viable regenerative therapy.

## Data Availability

No new data were created or analyzed in this study. Data sharing is not applicable to this article.
